# Bipolar disorder hospitalizations – a big data approach

**DOI:** 10.1192/j.eurpsy.2021.234

**Published:** 2021-08-13

**Authors:** M. Gonçalves-Pinho, J.P. Ribeiro, A. Freitas

**Affiliations:** 1 Department Of Psychiatry And Mental Health, Centro Hospitalar do Tâmega e Sousa, Penafiel, Portugal, Penafiel, Portugal; 2 2d4h, Center for Health Technology and Services Research (CINTESIS), Porto, Portugal

**Keywords:** Hospitalization, Big Data, bipolar disorder, Administrative Database

## Abstract

**Introduction:**

Bipolar Disorder (BD) is a mental disorder characterized by long hospitalizations and frequent need for acute psychiatric care. Hospitalizations represent a valuable quality of care indicator in BD.

**Objectives:**

The aim of this study was to describe a nationwide perspective of BD related hospitalizations and to use a BigData based approach in mental health research.

**Methods:**

We performed a retrospective observational study using a nationwide hospitalization database containing all hospitalizations registered in Portuguese public hospitals from 2008 to 2015. Hospitalizations with a primary diagnosis of BD were selected based on International Classification of Diseases version 9, Clinical Modification (ICD-9-CM) codes of diagnosis 296.xx (excluding 296.2x; 296.3x and 296.9x).

**Results:**

A total of 20,807 hospitalizations were registered belonging to 13,300 patients. 33.4% of the hospitalizations occurred in male patients and the median LoS was 16.0 days. Mean age was 47.9 years and male patients were younger(46.6 vs. 48.6; p< 0.001). 59 hospitalizations had a deadly outcome (0.3%). The most common cause of hospitalization in BD was the diagnosis code 296.4x (Bipolar I disorder, most recent episode (or current) manic) representing 34.3% of all hospitalizations, followed by the code 296.5x (Bipolar I disorder, most recent episode (or current) depressed) with 21.4%. The mean hospitalization charges were 3,508.5€ per episode, with a total charge of 73M€ in the 8-year period of this study.

**Conclusions:**

This is a nationwide study using BigData analysis giving a broad perspective of BD hospitalization panorama at a nationwide level. We found differences in hospitalization characteristics by gender, age and primary diagnosis.

**Disclosure:**

No significant relationships.

